# ZnO and TiO_2_ nanoparticles alter the ability of *Bacillus subtilis* to fight against a stress

**DOI:** 10.1371/journal.pone.0240510

**Published:** 2020-10-12

**Authors:** Elise Eymard-Vernain, Sylvie Luche, Thierry Rabilloud, Cécile Lelong

**Affiliations:** Université Grenoble Alpes, CNRS, CEA, IRIG, CBM UMR CNRS5249, Grenoble, France; VIT University, INDIA

## Abstract

Due to the physicochemical properties of nanoparticles, the use of nanomaterials increases over time in industrial and medical processes. We herein report the negative impact of nanoparticles, using solid growth conditions mimicking a biofilm, on the ability of *Bacillus subtilis* to fight against a stress. Bacteria have been exposed to sublethal doses of nanoparticles corresponding to conditions that bacteria may meet in their natural biotopes, the upper layer of soil or the gut microbiome. The analysis of the proteomic data obtained by shotgun mass spectrometry have shown that several metabolic pathways are affected in response to nanoparticles, n-ZnO or n-TiO_2_, or zinc salt: the methyglyoxal and thiol metabolisms, the oxidative stress and the stringent responses. Nanoparticles being embedded in the agar medium, these impacts are the consequence of a physiological adaptation rather than a physical cell injury. Overall, these results show that nanoparticles, by altering bacterial physiology and especially the ability to resist to a stress, may have profound influences on a “good bacteria”, *Bacillus subtilis*, in its natural biotope and moreover, on the global equilibrium of this biotope.

## Introduction

Nanoparticles are more and more present in manufactured products. They are mostly used for their particular physico-chemical properties, which have made them essential for many technological or industrial processes. In parallel, the scientific literature shows in an increasing manner that nanoparticles have significant and often deleterious impacts on the environment and consequently, on living organisms. Nanoparticles, and especially metal oxide nanoparticles, are often used and tested as antibacterial agents [1–6]. They are described as an alternative therapy against bacteria because, on one hand, the difficulties of bacteria to develop resistance to fight them and, on the other hand, for their moderate impacts on human cells at effective doses used to kill bacteria. This latter point is now more and more controversial considering direct [7–10] and indirect effects, for example effects on gut microbiome and therefore on pathophysiological response of host, of nanoparticles on the environment and animal or human health [11–17]. Their properties are mainly due to the damaging of the cell membrane, their accumulation/degradation and their interaction (direct or through their degradation products) with intracellular biomolecules, such as DNA or enzymes, inducing an oxidative stress by generating ROS, such as superoxides, hydrogen peroxide or hydroxyls radicals, leading to cell death. Their mechanisms to kill bacteria are more or less the same. But at sublethal doses, nanoparticles alter the cell metabolism of eukaryotic and prokaryotic cells, which can lead to the destabilization of a given ecosystem [18–20]. The natural biotope of *Bacillus subtilis* is the upper layer of soil, but it has also been found in the gut microbiome. It is also used to feed broiler chickens or fish to improve growth performance or immunology responses in absence of classical antibiotics [21–23]. It is also mixed with other bacteria to prepare probiotics for humans [24]. We have previously shown that the presence of nanoparticles in growth medium drastically alters the metabolism of *Bacillus subtilis*. In liquid conditions, nanoparticles (n-ZnO) induce the stringent response and major reorientations in the central metabolism [25]. To study the impact of nanoparticles during the formation of a biofilm in a contaminated soil, we studied the proteomic response of the ancestral strain *Bacillus subtilis* 3610, which is able to form a biofilm, contrary to the well known 168 laboratory strain. In this solid growth conditions, a soft agar medium, mimicking a biofilm, the competence, i.e. the first step of the horizontal gene transfer by the transformation process, is affected at a physiological level by the presence of nanoparticles in the growth culture medium [26].The impact on competence is dependent on the nature of the applied nanoparticles: n-TiO_2_ decrease the transformation efficiency of the *Bacillus subtilis* bacteria in biofilm growth conditions while n-ZnO increase it. Here, further analysis of proteomic data have highlighted the modification of the abundance of several proteins involved in different pathways linked to the defence against stresses: the stringent response, the oxidative stress or the response to methylglyoxal.

The presence of two nanoparticles, n-ZnO and n-TiO_2_, which have very different physico-chemical characteristics, has a deleterious impact on the physiological response of the bacteria cell to a stress, even at sublethal doses.

## Materials and methods

### Bacterial strain, culture media and chemicals

The *Bacillus subtilis* strain used was the 3610 strain (wild type) (personal gift, Dr Maria Laaberki). The medium was Luria-Bertani (LB): 10 g/l tryptone, 5 g/l yeast extract and 5 g/l NaCl. Cells were grown in Erlenmeyer flasks with shaking at 200 rpm at 37°C. The nanoparticles were from SIGMA: n-TiO_2_ (ref 700347, mixture 80:20 of anatase and rutile, 33–37 wt.% in water, <150 nm volume distribution by DLS, n-ZnO (ref 721077, 50% in water, <100 nm by DLS), and n-Ag (ref 758329, 5 wt% in ethylene glycol, <100 nm by TEM) were purchased as soluble dispersions. Their principal features have been described elsewhere [27,28].

### Biofilm agar plate preparation

The growth on soft solid agar LB medium was performed using a six well multi-well plate. Each well was filled with 7 ml of LB agar (10 g/l) containing (or none for control samples) a stress agent: 13 μg/ml n-TiO_2_, 17 μg/ml n-ZnO, 4.84 μg/ml ZnSO_4,_ 1 μg/ml silver lactate or 1 μg/ml n-Ag. The multi-well plate was dried overnight (15 h) at 37°C before being used.

### Biofilm growth conditions

A 3610 *Bacillus subtilis* culture grown overnight on liquid LB at 37°C was diluted to A_600nm_ = 0.1 in 10 ml of fresh LB medium and incubated at 37°C and 200 rpm until the A_600nm_ reached 0.7. 3 μl of this culture was inoculated in the middle of each well of the multi-well plates. The plates were then incubated at 30°C for 48 h. All the cells contained in a well were recovered using a sterile plastic inoculating loop. For the different assays described below, the bacteria were collected in PBS buffer.

All experiments were performed in triplicate (three independent growth cultures) and at least two technical replicates.

### Global proteomic analysis using PAST software

The relative abundance data provided by the proteomic data were used directly for global analysis using the PAST software suite [29]. In order to decrease the background of the analysis, only proteins showing at least one abundance change with a p-value<0.25 in a Welch test (TiO_2_ vs control, ZnO vs control or ZnSO_4_ vs control) were used [30]. Hierarchical clustering was selected as the analysis method for its insensitivity to missing or null values. In order to minimize the quantitative bias due to different protein abundances, the Gower distance (i.e. a normalized distance) was used to perform the clustering analysis.

### Indigo assay

Bacteria were grown, as described previously on LB agar (10 g/l) plate supplemented with BCIP plate: 12.5 μl of 100 mg/ml BCIP stock solution was added per LB agar plate. After resuspension in 500 μl of DMSO, the swarmed cells were incubated on a rotating wheel (Eppendorf® centrifuge 5430) during 15 minutes and then centrifuged at 13,000 rpm for 5 minutes. The indigo concentration was quantified by spectrophotometry (Jenway® 7315 spectrophotometer) at 636 and 620 nm. The indigo concentration was normalized by the total protein concentration measured using a Bradford assay.

### Methylglyoxal assay

5 mg/ml of 2,4-dinitrophenylhydrazine (DNPH) were diluted in 2M HCl to prepare the DNPH solution. 3610 *Bacillus subtilis* bacteria were grown in liquid LB medium until OD 600 reached 0.6. The growth was carried out during two hours in the presence of nanoparticles or Zn salts. The growth culture supernatant was collected after two successive centrifugations at 5,000 rpm for 15 minutes and stored at -20°C. To 200 μl of supernatant, 120 μl of DNPH solution were added and the resulting solution was incubated for 30 minutes at 30°C. 560 ul of 10% (w/v) NaOH were then added. After 10 min, the OD 500 was measured.

### Stress resistance assays

The bacteria resuspended in PBS buffer (see “Biofilm growth conditions” paragraph) were diluted in PBS to a final OD 600 = 1. They were then exposed to 1 mM of methylglyoxal or 5 mM of H_2_O_2_ at 37°C with shaking at 200 rpm. After 30 minutes, they were plated after appropriate dilutions on LB agar. CFU were counted after overnight incubation at 37°C.

All experiments were performed in triplicate (three independent growth cultures) and at least two technical replicates

### ppGpp assays

The ppGpp content was measured as previously described [31] using an on-off fluorescent probe. ppGpp extraction was performed on crude extracts of *Bacillus subtilis* obtained as described above in the “biofilm growth conditions” paragraph. Aliquots of the crude extract were used to measure the total protein quantity using the Bradford assay. The same quantity of each crude extract was precipitated with 10% formic acid for one hour on ice and cleared by centrifugation (13,000 rpm, for20 min). The pH of the supernatant was adjusted to 7.4 by the addition of N-ethylmorpholine (130 μl for a sample of 200 μl). Fluorescent probe preparation: 3 μM of oligonucleotide DNA (5’-GGC AGG TTG GGG TGA CTA AAA ACC CTT AAT CCC C-3’) and 90 μl of freshly prepared 1 mM silver lactate (Fluka BioChemika) were first mixed in 20 mM phosphate buffer (pH 6.6) for 5 min in a final volume of 1 ml, and kept protected from light at room temperature. After 20 min, 90 μl of freshly prepared 1 mM NaBH_4_ (Sigma-Aldrich) was quickly injected, thoroughly mixed, and the mixture was kept in the dark without further disturbance at 4°C overnight. Fluorescent detection: 20 μl of ppGpp extract was quickly mixed with the fluorescent probe (at a final concentration of 300 nM) in 20 mM phosphate buffer pH 7.4 in a final volume of 200 μl. The mixture was incubated 45 min at room temperature and protected from light before the first measurement (F_0_). Then, Cu^2+^ was added at a final concentration of 800 nM and the mixture was incubated 45 min at room temperature and protected from light before the second measurement (F). Fluorescent spectra were measured with a Tecan Infinite® M1000 instrument at a fluorescent excitation wavelength of 585 nm and fluorescent emission wavelength of 635 nm. The results show the average of a technical duplicate for each biological triplicate and are expressed as follow: [F/F_0_]stress condition normalized by [F/F_0_]without stress.

## Results

### Global analysis of the proteomic data

**Fig 1:**
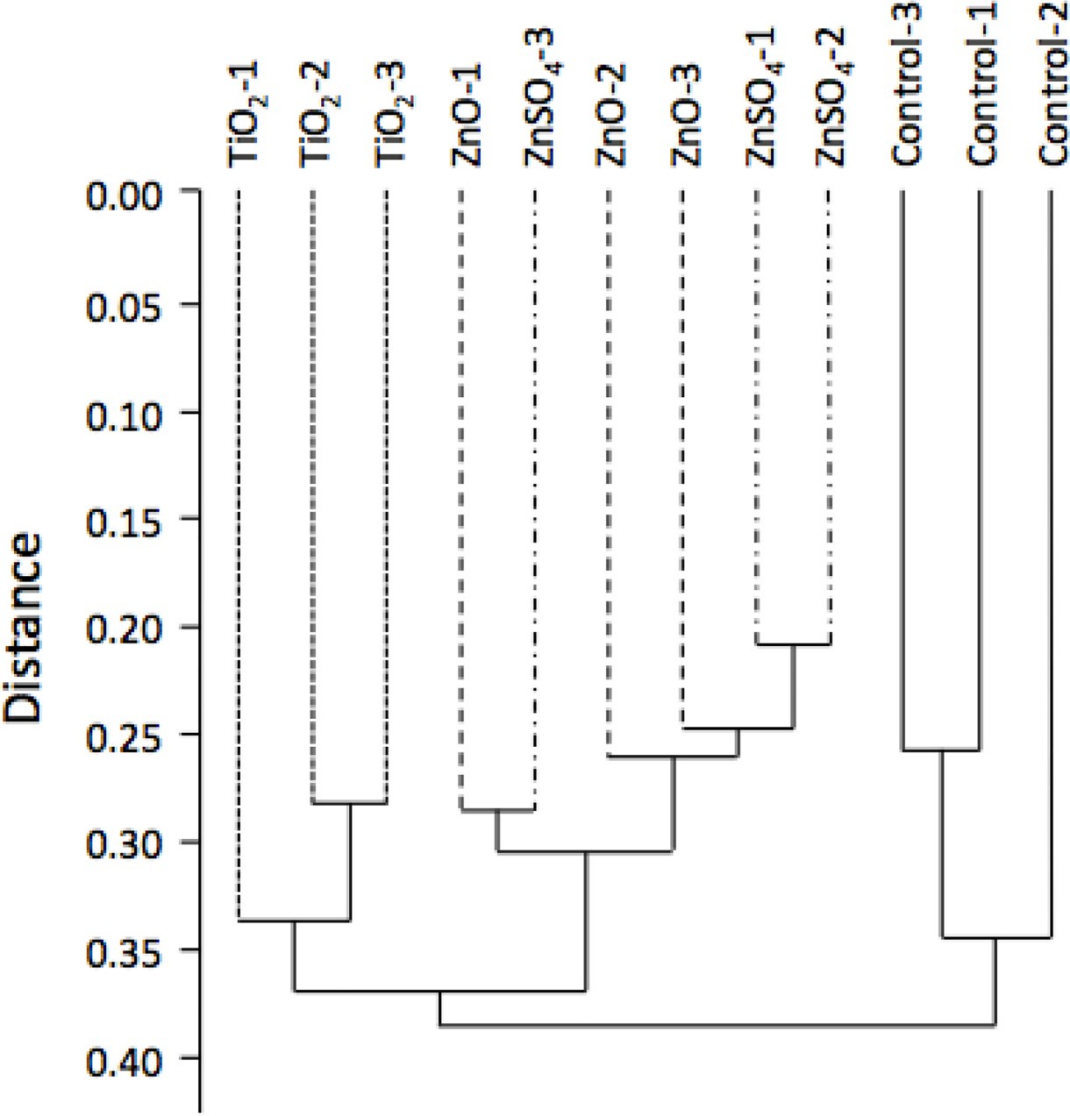
Global analysis of the proteomic experiment by hierarchical clustering. This tree indicates the similarity between the various experimental groups, where the higher the distance of the branching point between groups, the more dissimilar they are. Ctrl: unexposed bacteria; TiO_2_, ZnO, ZnSO_4_: bacteria exposed to nanoparticles or salts as described in material and methods.

As described by Eymard-Vernain [26], the proteomic responses to nanoparticles of 3610 *Bacillus subtilis* strain, grown on a soft agar LB plate to promote the swarming motion during the formation of biofilm, has been studied by shotgun mass spectrometry. The data are available at the PRIDE repository (data set identifier PXD006444). In order to assess the divergence between the different biological states investigated a global analysis by hierarchical clustering, specifically the abundance data provided by the proteomic study, was performed on 728 proteins. The results displayed in Fig 1, clearly show that the controls stood apart from all treated bacteria. The second division separated cells treated with titanium dioxide (n-TiO_2_) from cells treated with zinc. It was then impossible to separate cells treated with zinc ion (ZnSO_4_) from cells treated with zinc oxide (n-ZnO), showing that the effects were very similar. The analysis of the proteomic data has shown that several metabolic pathways are modified in presence of at least one nanoparticle.

### Methylglyoxal metabolism

**Fig 2:**
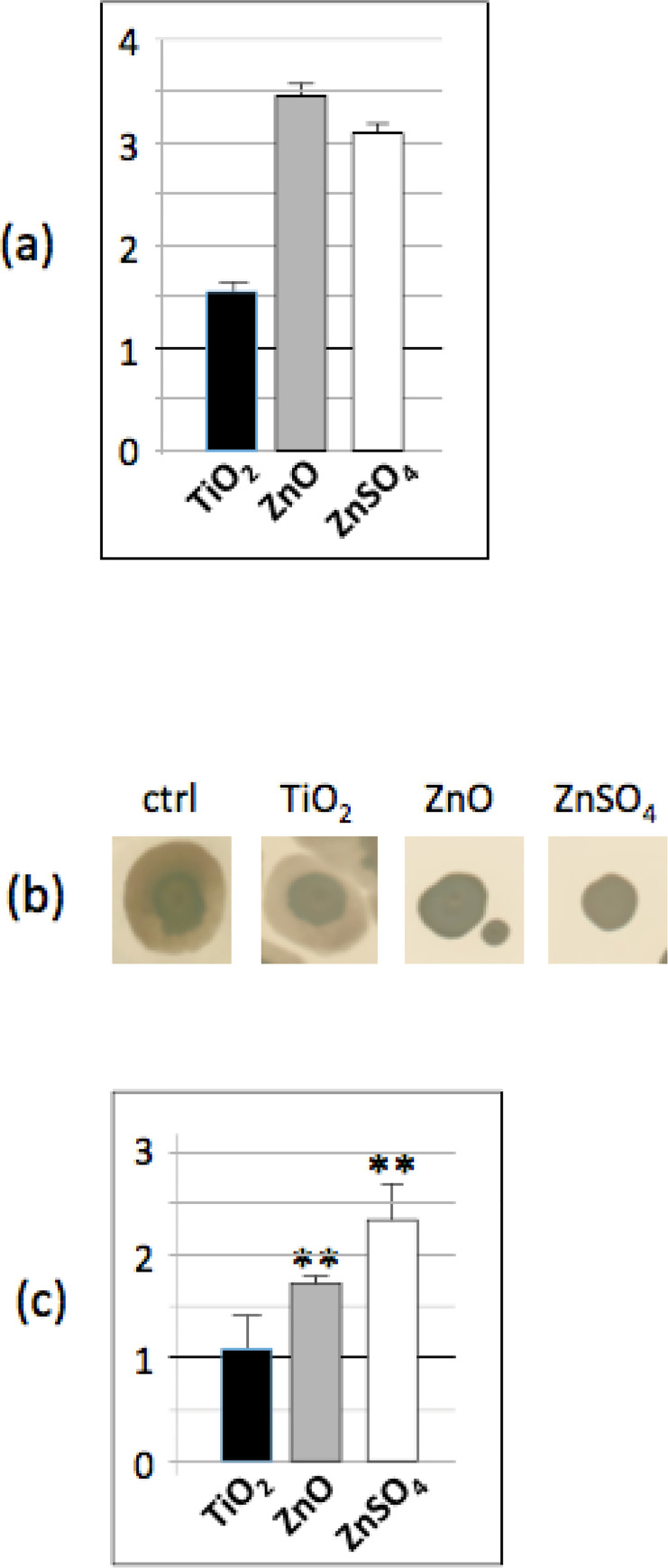
[Pi] is increased in presence of n-TiO_2_, n-ZnO and ZnSO_4_. (a) Abundance ratio of PhoA protein determined by proteomic analysis compared to the control: in black, ratio of the protein abundance in presence of n-TiO_2_/protein abundance in the control growth condition, in grey, in presence of n-ZnO and in white, in presence of ZnSO_4_ (b) Colony biofilms formed on LB soft agar supplemented with BCIP containing no nanoparticles, n-TiO_2_, n-ZnO or ZnSO_4_ salt after 48 of growth at 37°C (c) Indigo assays: indigo concentration in each growth condition is normalized by the indigo concentration in the control. Asterix ** indicate significant differences *p* < 0.005.

**Fig 3:**
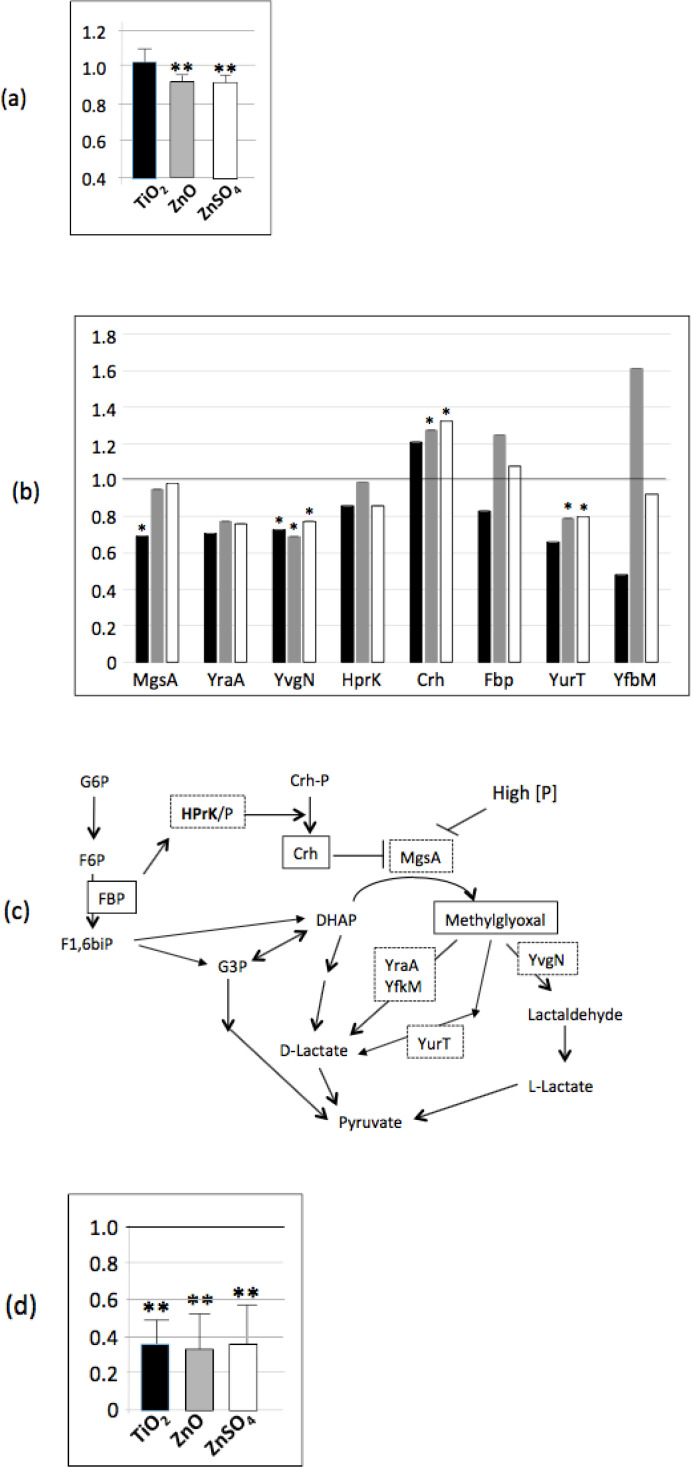
The methylglyoxal pathway is altered in presence of n-TiO_2_, n-ZnO and ZnSO_4_. (a) Methylglyoxal assays: methylglyoxal concentration in each growth condition is normalized by the methylglyoxal concentration in the control (b) abundance ratio of enzymes involved in the methylglyoxal pathway determined by proteomic analysis compared to the control: in black, ratio of the protein abundance in presence of n-TiO_2_/protein abundance in the control growth condition, in grey, in presence of n-ZnO and in white, in presence of ZnSO_4_ (c) Enzymes involved in methylglyoxal metabolism affected by the presence of n-TiO_2_, n-Zno and ZnSO_4_ in the medium and (d) Stress survival assays: cells previously exposed to n-TiO_2_, n-ZnO and ZnSO_4_ were exposed to 1 mM methylglyoxal for 30 min and after appropriate dilutions, they were plated on LB agar. The histogram shows the ratio of CFU of previously exposed cells/CFU of non exposed cells. Asterix * and ** indicate significant differences *p* < 0.05 and *p* < 0.005, respectively.

The abundance of the PhoA (alkaline phosphatase A) enzyme, which catalyses the production of inorganic phosphate (A phosphate monoester + H_2_O = > an alcohol + phosphate) is significantly increased (at least three fold), in the presence of n-ZnO or ZnSO_4_ salts and 1.5 fold when n-TiO_2_ (Fig 2A) is present. To assess the increase of the PhoA enzyme activity, the bacteria were grown on LB plates in biofilm growth conditions (see materials and methods), supplemented with BCIP (5-Bromo-4-chloro-3-indolyl Phosphate), in the same growth conditions used previously. Fig 2B and 2C show that the inorganic phosphate (Pi) concentration is significantly increased in the presence of n-ZnO and ZnSO_4_. In *Leishmania donovani* [32] or *Bacillus subtilis* [33], it has been shown that the modulation of the inorganic phosphate concentration is involved in the regulation of the methylglyoxal concentration. Methylglyoxal is a natural, but highly cytotoxic, by-product of glucose catabolism. Most of the living cells possess enzymatic machineries to detoxify methylglyoxal and avoid its accumulation in the cell. In parallel, methylglyoxal is secreted by the cells and can be detected in the medium. Thus, methylglyoxal production was measured in the supernatant of the *Bacillus subtilis* cultures exposed to nanoparticles in liquid LB medium during 2 hours: in presence of n-ZnO or ZnSO_4_, methylglyoxal excretion significantly decreased (Fig 3A). Interestingly, this measurement is in accordance with the proteomic results, which have shown a decreased abundance of YraA, YvgN and methylglyoxal synthase activity (MgsA), which are responsible of methylglyoxal degradation to D-Lactate, or methylglyoxal synthesis from lactaldehyde or DHAP (dihydroxyacetone phosphate), respectively (Fig 3B and 3C). This decreased abundance of enzymes necessary to detoxify the methylglyoxal is also supported by the decreased resistance to methylglyoxal of *Bacillus subtilis* cells previously exposed to n-TiO_2_ and n-ZnO nanoparticles or ZnSO_4_ salts (Fig 3D). Furthermore, the proteomic data have also shown that in the presence of n-ZnO or ZnSO_4_ salts, the abundance of the Fbp (fructose-1,6-bisphosphatase) and Crh (catabolite repression HPr-like protein) proteins increased, while those of the HprK (Hpr kinase/phosphorylase) and MgsA proteins decreased. In *Bacillus subtilis*, the regulation of production and detoxification of methylglyoxal is quite well understood (Fig 3C): briefly, the levels of DHAP and the phosphate concentration regulate its synthesis. High concentrations of DHAP promote MgsA and therefore methylglyoxal production, while high phosphate concentrations inhibit the MgsA enzyme. At the same time, the Crh protein, in response to the Fbp protein concentration, regulates MgsA expression: an increase of Fbp induced the dephosphorylation of HprK, which in turn promotes the dephosphorylated form of Crh, inhibiting MgsA. The proteomic data, including the increasing concentration of the inorganic phosphate, are in accordance with this known regulation pathway: increasing concentration of Zn in the medium induces high Piconcentration, which in turn impacts the methylglyoxal regulation pathway.

### Oxidative stress

**Fig 4:**
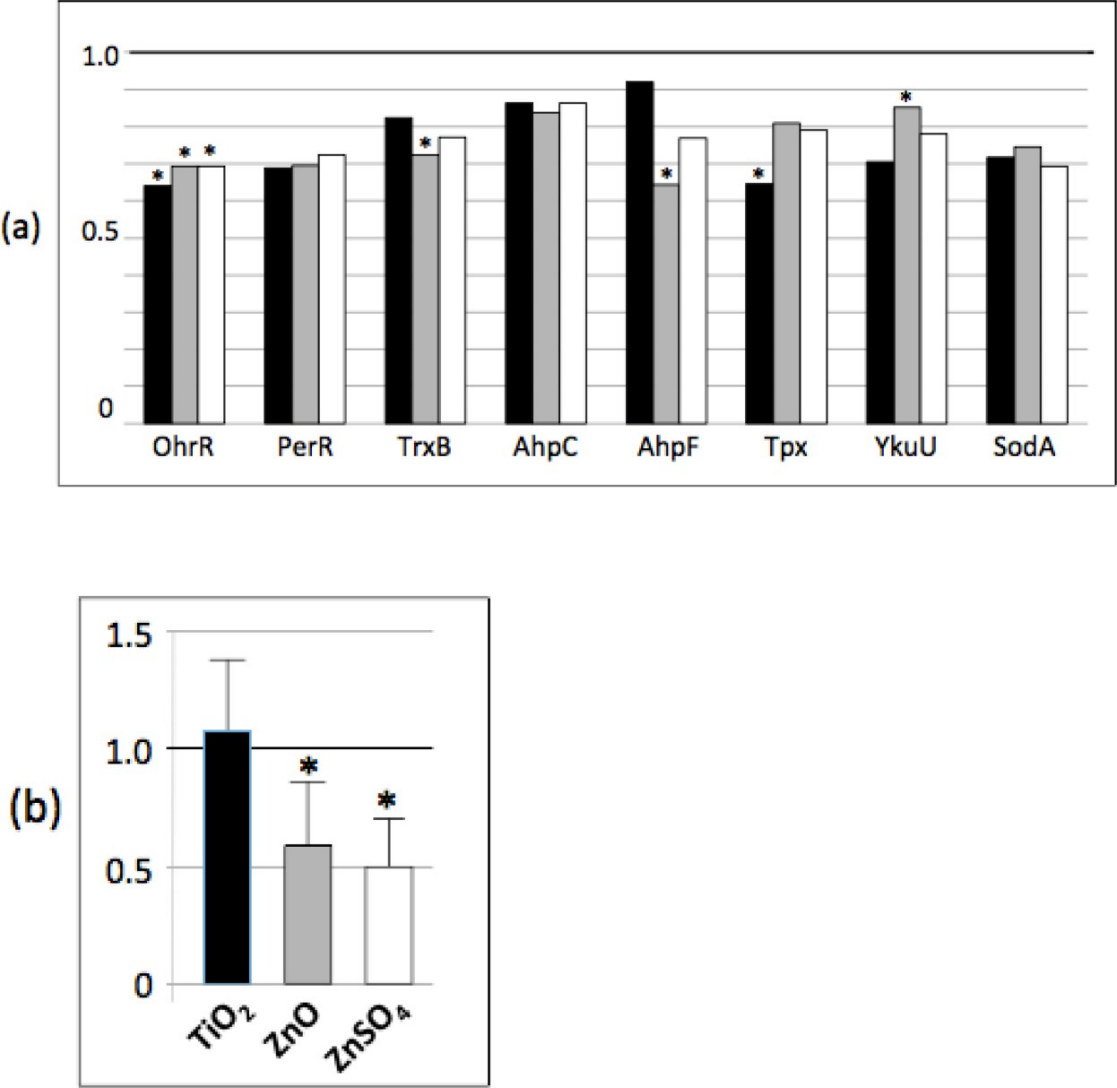
n-TiO_2_, n-ZnO and ZnSO_4_ affect the oxidative stress response. (a) Abundance ratio of proteins involved in the response to oxidative stress determined by proteomic analysis compared to the control: in black, ratio of the protein abundance in presence of n-TiO_2_/protein abundance in the control growth condition, in grey, in presence of n-ZnO and in white, in presence of ZnSO_4_ (b) Stress survival assays: cells previously exposed to n-TiO_2_, n-ZnO and ZnSO_4_ were exposed to 5 mM H2O2 during 30 mn and after appropriate dilutions, they were plate on LB agar. The histogram shows the ration of CFU of previously exposed cells/CFU for not exposed cells. Asterix * and ** indicate significant differences *p* < 0.05 and *p* < 0.005, respectively.

Mass spectrometry data have also shown that the abundance of several enzymes involved in the resistance to oxidative stress was decreased in the presence of n-TiO_2_, n-ZnO or ZnSO_4_ (Fig 4A, Table 1): OhrR, PerR, TrxB, AhpF, Tpx, YkuU and SodA. While the presence of n-TiO_2_ has no apparent effect on resistance to a secondary oxidative stress, *Bacillus subtilis* cells, previously exposed to n-ZnO or ZnSO_4_ were less resistant to H_2_O_2_ stress than the control, which is in accordance with the decreased abundance of enzymes involved in oxidative stress resistance (Fig 4A and 4B).

**Table 1:** Function of proteins highlighted in Fig 4A as described in the SubtiWiki database (http://subtiwiki.uni-goettingen.de/).

Name	Protein Function
**OhrR**	Organic hydroxyperoxide resistance transcriptional regulator
**PerR**	Peroxide operon regulator
**TrxB**	Thioredoxin reductase
**AhpF**	alkyl hydroperoxide reductase (large subunit)/NADH dehydrogenase
	Resistance against peroxide stress, PerR regulon
**Tpx**	Thiol peroxidase
**YkuU**	AhpA, alkyl hydroperoxide reductase
**SodA**	Superoxide dismutase

### Thiol metabolism

**Fig 5:**
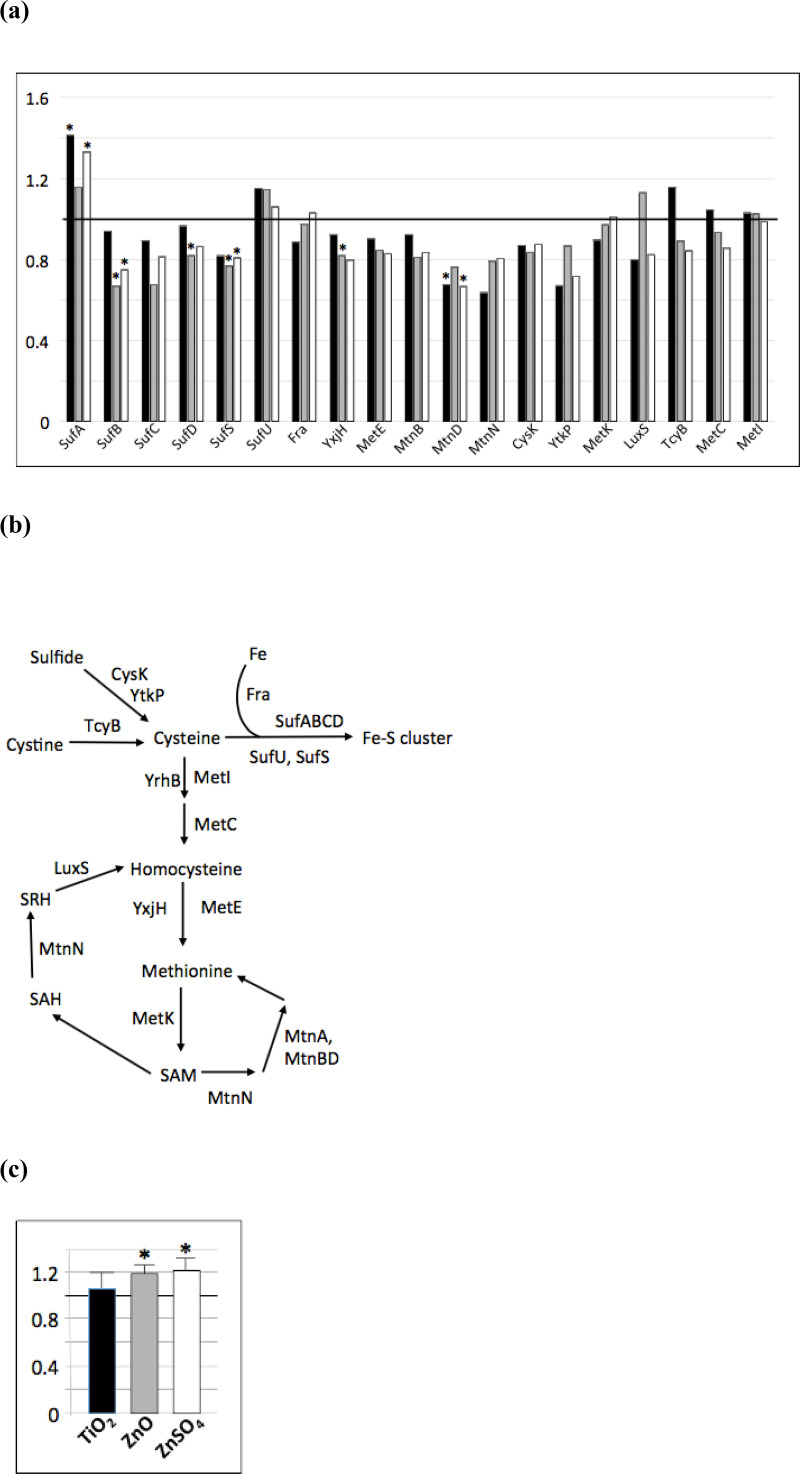
n-TiO_2_, n-ZnO and ZnSO_4_ affect the cysteine and methionine metabolisms. (a) Abundance ratio of proteins involved in cysteine and methionine metabolisms determined by proteomic analysis compared to the control: in black, ratio of the protein abundance in presence of n-TiO_2_/protein abundance in the control growth condition, in grey, in presence of n-ZnO and in white, in presence of ZnSO_4,_ (b) Enzymes involved in the cysteine and methionine metabolisms affected by the presence of n-TiO_2_, n-Zno and ZnSO_4_ in the medium and (c) Free SH assay as described in [25]. Asterix * indicates significant differences *p* < 0.05.

The abundance of 19 proteins involved in thiol metabolism was modified in presence of n-TiO_2_, n-ZnO or ZnSO_4_. The abundance of 16 of them decreased in at least one condition (Fig 5A and 5B). Most of these proteins are involved in cysteine and methionine catabolism (Fig 5B). In the presence of n-ZnO or ZnSO_4_, free thiol concentration increased significantly (Fig 5C). Low-molecular-weight thiols play a critical role for response to a stress. In *Bacillus subtilis*, three main low-molecular-weight thiols are related to cysteine and have been identified as cysteine itself, coenzyme-A and bacillithiol. The metabolism of bacillithiol is now well deciphered. The abundance of the main enzymes involved in the bacillithiol metabolism, BshA (N-acetyl-alpha-D-glucosamyl L-malate synthase), BshB1 (N-acetyl-alpha-D-glucosamyl L-malate deacetylase 1) and BshB2 (probable N-acetyl-alpha-D-glucosamyl L-malate deacetylase 2), was unmodified in the presence of nanoparticles or ZnSO_4_ (S1A Fig). Most of the enzymes involved in coenzyme A anabolism and detected during the proteomic analysis showed no significant modification of their abundance (e.g. IlvC, IlvD, PanB, YkpB, PanC, CoaA, CoaBC, and CoaD), except IlvD, which was significantly decreased in all conditions, and CoAD, which was significantly decreased in presence of n-ZnO (S1B Fig). IlvB, ilvH and CoAE were not detected. Concomitantly, it must be noticed that the abundance of AcsA, acetyl CoA synthase, and IolA, IolB, IolC, IolD, IolE, IolG, IolJ, coded by the operon *iolABCDEFGHIJ* and involved in the acetyl CoA and DHAP synthesis [34], were significantly decreased in presence of n-ZnO or ZnSO_4_ (S2A and S2B Fig). This observation can be correlated to the decreased amount of methylglyoxal in cells, due to DHAP being the substrate of MgsA to produce methylglyoxal, in response to n-ZnO, n-TiO_2_ or ZnSO_4_.

### Stringent response

**Fig 6:**
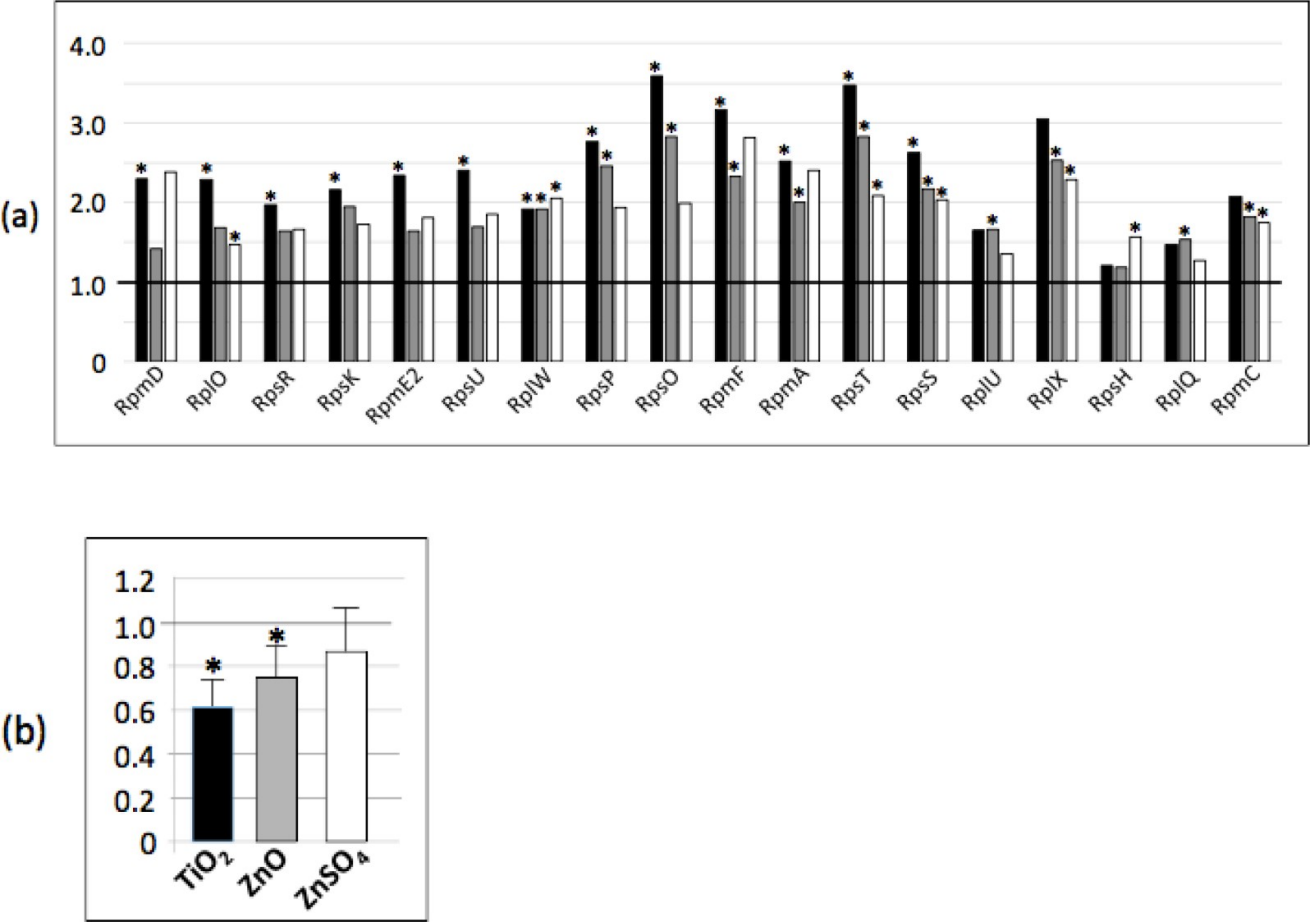
n-TiO_2_, n-ZnO and ZnSO_4_ affect the stringent response. (a) Abundance ratio of ribosomal proteins determined by proteomic analysis compared to the control: in black, ratio of the protein abundance in the presence of n-TiO_2_/protein abundance in the control growth condition, in grey, in presence of n-ZnO and in white, in presence of ZnSO_4_ (b) ppGpp assay performed as described in [25]. Asterix * and ** indicate significant differences *p* < 0.05 and *p* < 0.005, respectively.

In the study published by Luche and coll. [25],where the bacterial cells were grown in liquid medium, we showed that n-ZnO and ZnSO_4_ affected the abundance of at least nine proteins involved in the stringent response. In a solid medium growth condition, with n-ZnO, TiO_2_ and ZnSO_4_, the abundance of at least 17 proteins increased, compared to the control condition (Fig 6A and Pride repository PXD006444). In stressful conditions, the translation process, including the expression of ribosomal proteins themselves, is controlled by the stringent response. The intracellular (p)ppGpp (the guanosine penta- or tetra-phosphate) level is the central regulator of the stress response. Three main enzymes control its concentration, i.e. RelA, SasA and SasB. SasA and SasB were not detected by the proteomic analysis while the abundance of RelA was unchanged along the different conditions. However, the (p)ppGpp assay showed a significantly decreased amount of (p)ppGpp in the presence of nanoparticles, either n-ZnO or n-TiO_2_ (Fig 6B). This decrease is correlated to the increased abundance of ribosomal proteins [35].

## Discussion

The analysis of the proteomic data has shown that, for *Bacillus subtilis* in biofilm growth conditions, the abundance of many proteins involved in stress response is altered by the presence of nanoparticles in the medium. As already described [26], when nanoparticles are embedded in the soft agar medium, the observed phenotypes are the consequences of an alteration of physiological state of the bacteria and not the consequence of a direct physical interaction. Moreover, *Bacillus subtilis* cells are more sensitive to a secondary stress and particularly oxidative stress after a long exposure to nanoparticles or ZnSO_4_ salts. The zinc ions liberated by n-ZnO and ZnSO_4_ are highly likely to be the main agent responsible for the alteration of the stress response but the n-TiO_2_ particles, also showed a significant impact, even if more moderate.

As previously described [26], the presence of n-ZnO in the medium causes the release of Zn ions at a concentration greater than that required for optimal growth. This excessive zinc concentration leads to the activation of the competence process [26]. The nanoparticles and the ZnSO_4_ being embedded in agar medium, it is not possible to quantify the relative concentration of dissolved Zn^2+^ from n-ZnO in the medium. But in a previous publication [26], we have shown that the intracellular zinc concentration was significantly increased with the same magnitude order, in both conditions, n-ZnO and ZnSO_4_ salt, while the total quantity of zinc introduced in the medium is ten times higher with n-ZnO than with ZnSO_4_. Furthermore, Li and colleagues [36] have shown, that most Zn^2+^ ions are complexed with amino acids resulting in a dramatic decrease of the n-ZnO toxicity. In our conditions, where n-ZnO is embedded in agar plate, the results let suppose that n-ZnO is not totally dissolved in a solid and complex biological medium. Here, we show that the presence of n-ZnO, ZnSO_4_ but also of n-TiO_2_ in the LB agar medium induce, in one hand, the inhibition of the stringent response and of the oxidative stress response, and, on the other hand, decreases the free thiol concentration and methylglyoxal metabolism, leading to a decrease in the extracellular concentration of methylglyoxal. Furthermore, the regulatory protein Spx is involved in the regulation of most of these physiological processes, but in an opposite manner [37,38]. In fact, Spx is a pleiotropic regulator of *Bacillus subtilis* which, by interacting with the alpha subunit of the RNA polymerase, can inhibit or activate the transcription of many operons in response to disulphide stress. For example, it up-regulates the methylglyoxal metabolism while it down-regulates competence, translation, or the thiol oxidative stress response or the [38–40]. It also plays an important role in stress adaptation: for example, cells lacking *spx* are more sensitive to antibiotics or heat-shock [41,42]. Spx level is finely controlled at several levels, at least at transcriptional and post-translational levels. Moreover, its regulatory activity, i.e., its interaction with the RNAP, is under the control of its redox state, Spx_red_ < = > Spx_ox_. An excess of Spx is also deleterious for competence or for sporulation [43]. *Bacillus subtilis* possess two Spx paralogs: MgsR and YusI. Neither Spx nor MgsR were detected during the proteomic shotgun analysis. The YusI protein was detected, but its level was unmodified in response to nanoparticles or ZnSO_4_ salts. However, many genes described in the results section belong to the Spx regulon (*mgsA*, *tpx*, *perR*, *ahpF*, *trxB*, *cysK*, *mtnN*), suggesting a potential implication of the Spx and Spx-like main regulators.

**Fig 7:**
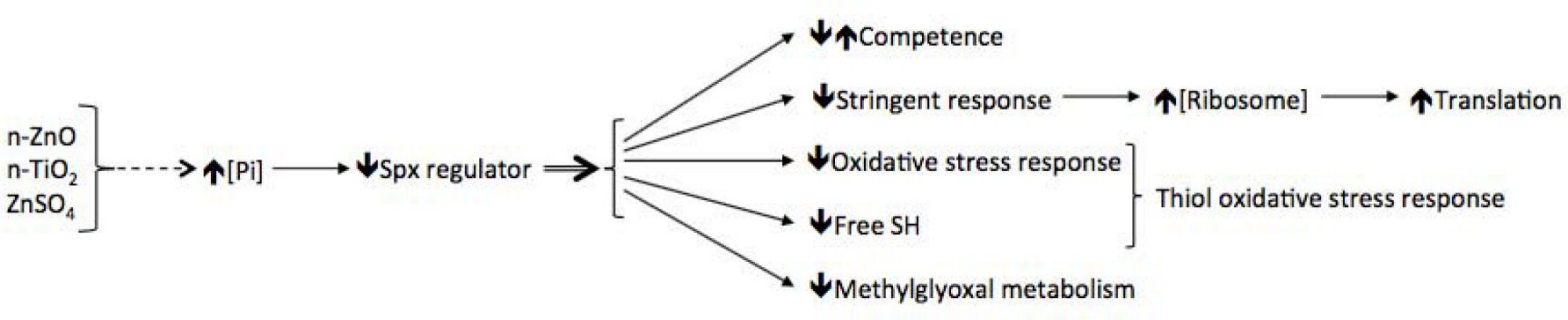
Probable involvement of the Spx regulator. Impact of n-TiO_2_, n-ZnO and ZnSO_4,_ in a soft agar growth condition, on the *Bacillus subtilis* stress response.

Auger and collaborators [44] and our group [25] have shown that an excess of Zn ions in liquid growth conditions (LB) and with a short exposure, leading to the dissolution of ZnO nanoparticles, induces an oxidative stress, to which cells respond by the expression of several enzymes or processes: catalase, ferritin, stringent response or Spx regulator. Here, in soft agar growth conditions, where the bacterial cells were exposed for a long time, but not physically in contact with an excess of Zn (or to n-TiO_2_), we observe an opposite phenotype. It is remarkable to notice that even in the presence of n-TiO_2_, which has minimal impact at a macroscopic level (e.g., the aspect of the swarming colony) when compared to n-ZnO [26], the abundances of many proteins, identified and quantified by shotgun proteomics, are modified in the presence of both nanoparticles. Knowing that *spx* transcription is stimulated by phosphate starvation [45], we proposed a schematic pathway to explain the physiological response of *Bacillus subtilis* to the nanoparticles in soft agar growth conditions (Fig 7). The nanoparticles induced an increased [Pi], the Spx protein expression and/or activity was repressed, and thereby lead to the inhibition of the stringent response, the oxidative stress response, the thiol stress response and the methylglyoxal metabolism, while the competence was increased.

The growth conditions tested here, i.e., weakly toxic doses of nanoparticles for a long period in a biofilm “like” condition, mimic the conditions met by the bacteria in a natural context, i.e., around the root of plants in the rhizosphere or in the gut microbiome. Methylglyoxal has been found in many food and beverages, so that bacteria of the gut microbiome may be regularly exposed to methylglyoxal [46]. In addition, methylglyoxal is produced by macrophages as part of the response to invasion by pathogenic bacteria [47]. An increase in methylglyoxal-associated DNA damage in response to n-ZnO has also been documented in mammalian cells [48]. The response of eukaryotic and prokaryotic cells to the presence of n-ZnO in the growth medium is opposite: n-ZnO induces the production of methylglyoxal in the macrophage, whereas it decreases in *Bacillus subtilis*.

*Bacillus subtilis*, in all of its biotopes, rhizosphere or gut, is a “good” bacterium. In the rhizosphere, it contributes to plant development and protection by the secretion of antifungal or antivirus agents, such as surfactin or fengycin lipopeptides for example. It is also used as a probiotic to promote the growth of broiler chicken or shrimp. Several studies have shown that the co-administration of nanoparticles and probiotics has no or few effect on the microbiome composition or activity [49,50]. But chronic oral absorption of nanoparticles, as a consequence of nanoparticles in food additives, disturbs the gut microbiome equilibrium and may be responsible for several chronic diseases [51]. Most of the time, the effect of the nanoparticles is dose and time-dependent. In vivo and in vitro studies have shown that even at low and/or sublethal doses, and with no direct contact with the living cells, as in the present study, the nanoparticles alter cell metabolism, and therefore may modify most of considered biotopes. All these results raise the question of the impact of nanoparticles on a “good bacteria” in its natural biotope and moreover, on the global equilibrium of this biotope.

## Supporting information

S1 Fig (a) Abundance ratio of proteins involved in bacillithiol synthesis determined by proteomic analysis compared to the control: in black, ratio of the protein abundance in the presence of n-TiO_2_/protein abundance in the control growth condition, in grey, in presence of n-ZnO and in white, in presence of ZnSO_4_ (b) Abundance ratio of proteins involved in CoA anabolism and *iol* operon, determined by proteomic analysis compared to the control: in black, ratio of the protein abundance in the presence of n-TiO_2_/protein abundance in the control growth condition, in grey, in presence of n-ZnO and in white, in presence of ZnSO_4._ Asterix * indicates significant differences *p* < 0.05.
(TIF)

S2 Fig (a) Scheme of the inositol metabolism (b) Abundance ratio of proteins coding by the *iol* operon, determined by proteomic analysis compared to the control: in black, ratio of the protein abundance in the presence of n-TiO_2_/protein abundance in the control growth condition, in grey, in presence of n-ZnO and in white, in presence of ZnSO_4_. Asterix * indicatse significant differences *p* < 0.05.
(TIF)
