# Supplementary figures and images for: ZnO and TiO2 nanoparticles alter the ability of Bacillus subtilis to fight against a stress
Source: PLoS One. 2020 Oct 12;15(10):e0240510. doi: 10.1371/journal.pone.0240510 (PMC7549824; doi:10.1371/journal.pone.0240510)

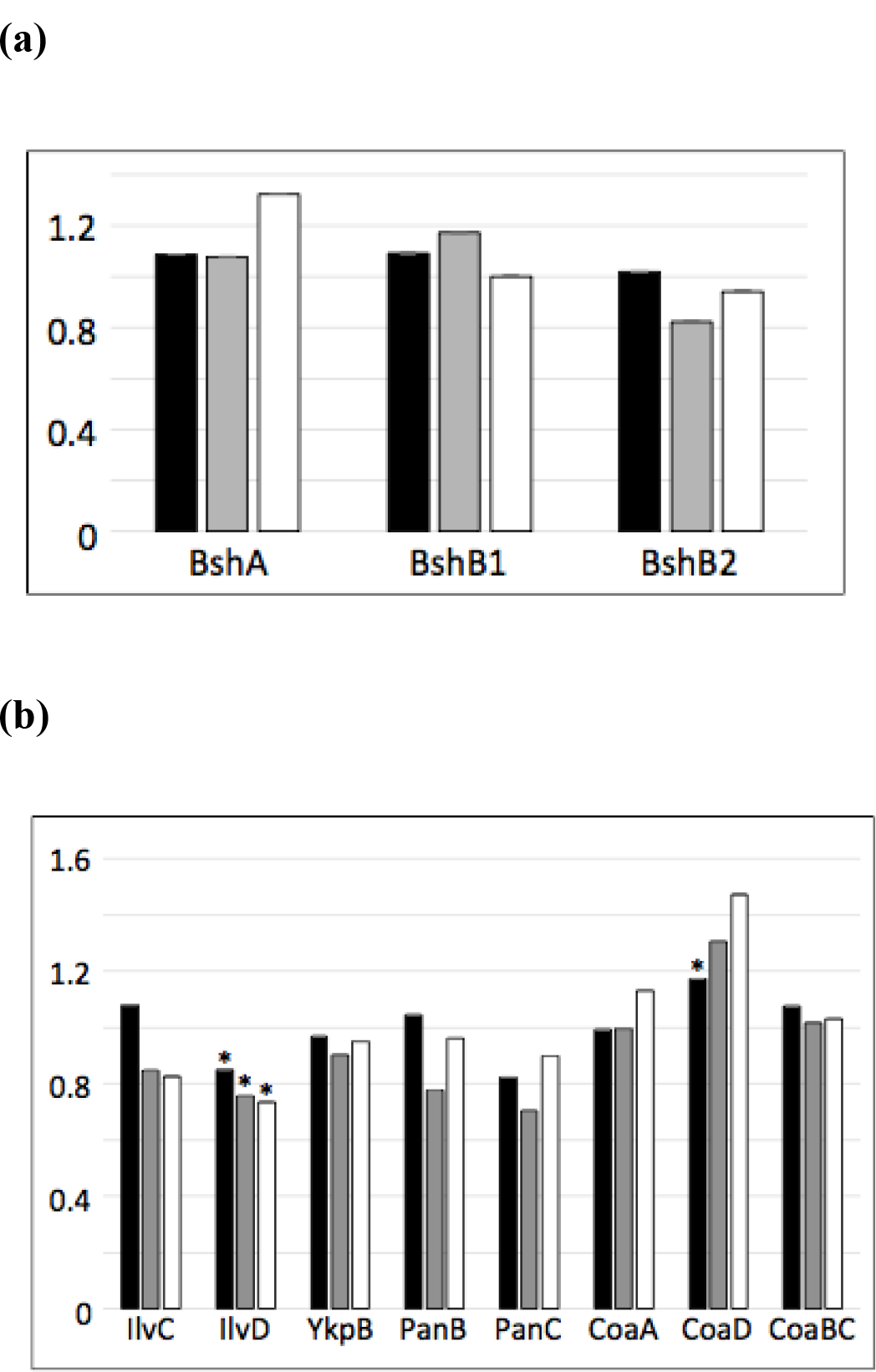

Supplement: S1 Fig — (a) Abundance ratio of proteins involved in bacillithiol synthesis determined by proteomic analysis compared to the control: in black, ratio of the protein abundance in the presence of n-TiO2/protein abundance in the control growth condition, in grey, in presence of n-ZnO and in white, in presence of ZnSO4 (b) Abundance ratio of proteins involved in CoA anabolism and iol operon, determined by proteomic analysis compared to the control: in black, ratio of the protein abundance in the presence of n-TiO2/protein abundance in the control growth condition, in grey, in presence of n-ZnO and in white, in presence of ZnSO4. Asterix * indicates significant differences p < 0.05. (TIF) [file pone.0240510.s001.tif]

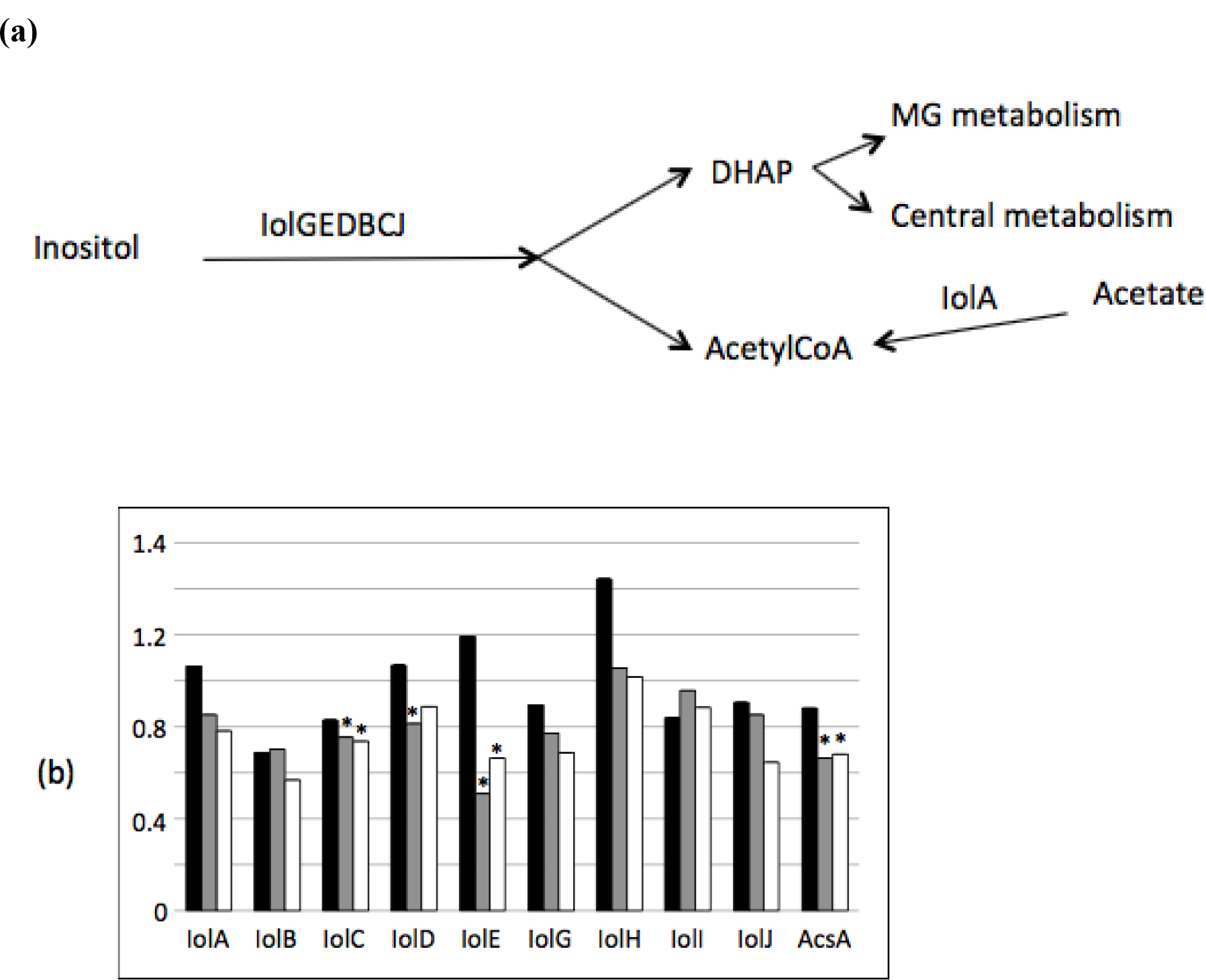

Supplement: S2 Fig — (a) Scheme of the inositol metabolism (b) Abundance ratio of proteins coding by the iol operon, determined by proteomic analysis compared to the control: in black, ratio of the protein abundance in the presence of n-TiO2/protein abundance in the control growth condition, in grey, in presence of n-ZnO and in white, in presence of ZnSO4. Asterix * indicatse significant differences p < 0.05. (TIF) [file pone.0240510.s002.tif]
